# The glycosylation status of MHC class I molecules impacts their interactions with TAPBPR

**DOI:** 10.1016/j.molimm.2021.09.007

**Published:** 2021-11

**Authors:** F. Tudor Ilca, Louise H. Boyle

**Affiliations:** Department of Pathology, University of Cambridge, Cambridge, CB2 1QP, UK

**Keywords:** Antigen processing and presentation, TAPBPR/TAPBPL, Peptide exchange, Peptide editing, MHC, Glycosylation

## Abstract

Glycosylation plays a crucial role in the folding, structure, quality control and trafficking of glycoproteins. Here, we explored whether the glycosylation status of MHC class I (MHC-I) molecules impacts their affinity for the peptide editor, TAPBPR. We demonstrate that the interaction between TAPBPR and MHC-I is stronger when MHC-I lacks a glycan. Subsequently, TAPBPR can dissociate peptides, even those of high affinity, more easily from non-glycosylated MHC-I compared to their glycosylated counterparts. In addition, TAPBPR is more resistant to peptide-mediated allosteric release from non-glycosylated MHC-I compared to species with a glycan attached. Consequently, we find the glycosylation status of HLA-A*68:02, -A*02:01 and –B*27:05 influences their ability to undergo TAPBPR-mediated peptide exchange. The discovery that the glycan attached to MHC-I significantly influences the affinity of their interactions with TAPBPR has important implications, on both an experimental level and in a biological context.

## Highlights

• The interaction between TAPBPR and MHC-I is stronger when MHC-I lacks a glycan.
• TAPBPR dissociates peptides more easily from non-glycosylated MHC-I.
• Glycosylation status of MHC-I influences their ability to undergo peptide exchange.
• MHC-I trafficking through the secretory pathway will impact TAPBPR functionality.

## 1 Introduction

Protein glycosylation is critical for numerous biological processes. By influencing interactions with chaperones and enzymes, the glycan attached to glycoproteins affects quality control, trafficking to the plasma membrane, protection from degradation as well as influencing protein structure, stability and spacing (Rudd et al., 2001; Wolfert and Boons, 2013). Sugar attachments can serve as ligands for receptor and can also have a significant influence on protein-protein interactions through their size and hydrophobicity (Rudd et al., 2001; Wolfert and Boons, 2013). For human MHC class I (MHC-I) molecules a single conserved N-linked glycan is located on an asparagine residue found at position 86 (Parham et al., 1977). The sugar moiety on MHC-I governs its interaction with a number of key components of the antigen processing and presentation pathway. For example, when the glycan attached to MHC-I is Glc_1_Man_9_GlcNAc_2_, this facilitates its interaction with the lectin chaperones calnexin and calreticulin, thus aiding MHC-I folding and assembly (Ware et al., 1995; Vassilakos et al., 1998). Through its interaction with calreticulin and ERp57, the glycan attached permits peptide-receptive MHC-I to bind to the peptide editor tapasin and consequently be incorporated into the peptide loading complex (PLC) (Radcliffe et al., 2002; Wearsch and Cresswell, 2008; Rizvi et al., 2011; Wearsch et al., 2011). Thus, the glycosylation status of MHC-I molecules promotes their acquisition of antigenic cargo for presentation to the immune system. Furthermore, the egress of MHC-I from the ER/cis-Golgi to the plasma membrane is also controlled by the sugar moiety attached; removal of the terminal-glucose residue from peptide loaded MHC-I by glucosidase I/II initiates egress from the ER/cis-Golgi, while the subsequent reglucosylation of molecules deemed unsuitable for export by UDP-glucose: glycoprotein glucosyltransferase 1 (UGT1) causes ER retention of MHC-I by promoting rebinding to calreticulin and consequently reincorporation into the PLC (Wearsch and Cresswell, 2008; Wearsch et al., 2011; Zhang et al., 2011).

In addition to tapasin’s essential role in loading and selecting peptide onto MHC-I (Williams et al., 2002; Howarth et al., 2004; Chen and Bouvier, 2007; Wearsch and Cresswell, 2007), the tapasin-related protein TAPBPR also functions as an MHC-I peptide editor that shapes the peptide repertoire presented on cells (Hermann et al., 2015; Morozov et al., 2016; Ilca et al., 2018a). The discovery of TAPBPR (Teng et al., 2002; Boyle et al., 2013) has helped revealed new mechanistic insight regarding peptide selection on MHC-I. Crystal structures of TAPBPR:MHC-I complexes have shown TAPBPR widens the peptide-binding groove of MHC-I and demonstrated molecular changes at the MHC-I F pocket in the process of peptide editing (Thomas and Tampe, 2017; Jiang et al., 2017). Recent work suggests a loop region of TAPBPR, composed of residues 22–35, is a key functional region in performing peptide editing and is thought to influence peptide selection through insertion into the MHC-I peptide binding grove (Ilca et al., 2018b; Sagert et al., 2020), a hypothesis supported by proximity of this region in the crystal structures (Thomas and Tampe, 2017; Jiang et al., 2017). In addition, NMR-based interrogation of the TAPBPR:MHC-I complex has confirmed that TAPBPR stabilises peptide-receptive MHC-I and suggests peptide selection is mediated by an allosteric release mechanism (McShan et al., 2018). Whether all MHC-I molecules undergo TAPBPR-mediated peptide exchange is still unclear. Recently, polymorphisms in MHC-I were found to impact their ability to be edited by TAPBPR, and demonstrated members of the HLA-A2 and –A24 superfamily, were particularly receptive to TAPBPR-mediated peptide editing (Ilca et al., 2019).

Interestingly, TAPBPR plays a role in influencing the sugar moiety attached to MHC-I. By functioning as a bridge between MHC-I and UGT1, TAPBPR helps promotes MHC-I reglucosylation and recycling back to the PLC (Neerincx et al., 2017). Following this discovery, we explored whether the interaction between TAPBPR and MHC-I was dependent on the glycan attached to MHC-I. To this end, we tested whether TAPBPR could bind to MHC-I molecules in which the NxS/T motif was mutated, thus eliminating N-linked glycosylation. We found the interaction between TAPBPR and MHC-I occurred in a glycan-independent manner, that is to say TAPBPR could bind to MHC-I either with or without an N-linked glycan attached (Neerincx and Boyle, 2019a). Thus, in contrast to the narrow glycan specificity of tapasin, TAPBPR has the potential to associate with MHC-I with a broad diversity of oligosaccharide attachments.

Our previous data hinted that the interaction between TAPBPR:MHC-I may be more stable when MHC-I lacked N-linked glycosylation (Neerincx and Boyle, 2019a). While on a physiological level it is unlikely that TAPBPR will access glycan-deficient MHC-I, on an experimental level, many studies exploring the interaction between TAPBPR and MHC-I have utilised MHC-I lacking a glycan attachment by using bacterially expressed MHC-I. These include affinity and structural studies of the TAPBPR:MHC-I complex (Morozov et al., 2016; Thomas and Tampe, 2017; Jiang et al., 2017; McShan et al., 2018) as well as those utilising *in vitro* peptide editing assays to explore TAPBPR function (Hermann et al., 2015; Morozov et al., 2016; Sagert et al., 2020; Neerincx et al., 2017). Thus, it is important to determine whether the glycosylation status of MHC-I impacts its affinity for TAPBPR in order to determine how reflective the use of non-glycosylated MHC-I is to the *in vivo* scenario. Here, we reveal that the interaction between TAPBPR and MHC-I is stronger when MHC-I lacks a glycan and that this consequentially impacts the efficiency of TAPBPR to perform peptide editing.

## 2 Materials and methods

### 2.1 Constructs and cell lines

The panel of HeLaM cell lines, deficient of HLA-A,B,C heavy chains (HeLaM-HLA-ABC^KO^) and reconstituted with each individual variant (WT, N86Q and N88R) of each individual MHC-I allotype were used in this study (HLA-A*02:01, HLA-A*68:02 and HLA-B*27:05) (Neerincx and Boyle, 2019a). All cell lines were cultured and maintained in Dulbecco's Modified Eagle's medium (DMEM; Sigma-Aldrich, UK), supplemented with 10 % fetal calf serum (Gibco, Thermo Fisher Scientific), 100 U/mL penicillin and 100 μg/mL streptomycin (Gibco, Thermo Fisher Scientific) at 37 °C with 5% CO_2_. Each cell line tested negative for mycoplasma (MycoAlert, Lonza, UK). To up-regulate MHC-I expression, as well as other components of the MHC-I antigen processing and presentation pathway, cells were treated with 200 U/mL of IFN-γ (Peprotech) for 48–72 h, prior to each experiment.

### 2.2 Expression and purification of soluble TAPBPR protein

Secreted versions of TAPBPR^WT^ and TAPBPR^TN5^ proteins, consisting of their corresponding lumenal domains alone, with an introduced C-terminally His_6_ tag, were expressed in HEK 293 T cells using a PiggyBac expression system and purified by Ni^2+^-affinity chromatography, as previously described (Ilca et al., 2018a).

### 2.3 MHC-I binding peptides

The fluorescently-labelled peptides specific for HLA-A*68:02 used in this study were ETVSK*QSNV and YVVPFVAK*V (K* = a lysine residue labelled with 5-carboxytetramethylrhodaime [TAMRA]), the ones specific for HLA-A*02:01 were NLVPK*VATV and YLLEK*LWRL and the one for HLA-B*27:05 was SRYWK*IRTR, as previously used (Ilca et al., 2018a). All peptides were purchased from Peptide Synthetics, UK.

### 2.4 Antibodies

TAPBPR was detected using the mouse mAb PeTe-4, raised against the native conformation of TAPBPR (Boyle et al., 2013) while MHC class I molecules on the cell surface were detected using the pan-HLA class I-specific mouse mAb W6/32, produced in house (Barnstable et al., 1978). A FITC-labelled goat anti-mouse secondary antibody was also used (Invitrogen Probes, Thermo Fisher Scientific)

### 2.5 Treatment of cells with brefeldin A

BFA decay experiments were performed as previously described (Ilca et al., 2019). Briefly, HeLa-HLA-ABC^KO^ cells, reconstituted with individual MHC-I allotypes were IFN-γ-stimulated for 48 h and then treated with 10 μg/mL BFA (Sigma-Aldrich) diluted in DMEM. Cells were then harvesting and the MHC class I levels at the surface were measured by flow cytometry, by staining with the W6/32 antibody.

### 2.6 TAPBPR binding and peptide exchange assays on cell surface MHC-I

The binding of either soluble TAPBPR alone to cells or of fluorescently-labelled peptides to surface expressed MHC-I molecules in the presence of TAPBPR were performed as previously described (Ilca et al., 2018a). Briefly, cells seeded on 12-well plates were treated with either TAPBPR alone for 30 min at 37 °C, at different concentrations, or with fluorescently-labelled peptides, in the presence or absence of TAPBPR, for different time periods. Excess unbound TAPBPR and/or peptides were then washed and the levels of TAPBPR/peptide binding were quantified by flow cytometry.

### 2.7 Flow cytometry

Following trypsinization, cells were washed in 1x PBS containing 1% bovine serum albumin and placed on ice. Cells were subsequently stained with either PeTe-4, used for TAPBPR detection, or W6/32, used for peptide-bound MHC-I detection, for 30 min on ice. For each experiment, staining with an isotype control antibody was included as well. The primary antibodies were then detected by incubation with the Alexa 647-labelled goat anti-mouse secondary antibody (Invitrogen, Thermo Fisher Scientific). Following another 3 rounds of washing, the fluorescence levels were detected using an Attune analyzer (Invitrogen, Thermo Fisher Scientific).

### 2.8 TAPBPR/peptide dissociation assays from cell surface MHC-I

When measuring peptide-mediated TAPBPR dissociation from MHC-I molecules, TAPBPR was added to cells at 1 μM for 30 min. Excess TAPBPR was then removed by thorough washing and high-affinity peptide was then added to cells for 30 min at different concentrations. The levels of TAPBPR dissociation were measured by flow cytometry, as a reduction in the bound TAPBPR fraction upon peptide treatment.

When measuring TAPBPR-mediated peptide dissociation from MHC-I, 10 nM fluorescent peptides were first added to cells in the presence of TAPBPR, for an HLA I-specific incubation period. Subsequently, excess peptides and TAPBPR were removed by washing and peptide dissociation was measured by flow cytometry, as a reduction in the fraction of bound peptide upon TAPBPR treatment.

## 3 Results

### 3.1 MHC-I lacking a glycan display an enhanced ability to associate with TAPBPR

Our previous studies hinted that the TAPBPR:MHC-I interaction may be stronger when MHC-I lacks a glycan (Neerincx and Boyle, 2019a). However, the experimental system previously used was not optimised to explore strength of binding. Furthermore, removal of MHC-I glycan resulted in consequential knock-on effects on both competitive interactions (e.g. with tapasin) and MHC-I recycling within the intracellular environment, resulting in us being unable to determine specifically whether affinity was indeed altered (Neerincx and Boyle, 2019a; Neerincx and Boyle, 2019b). Having subsequently developed novel assays to investigate TAPBPR binding to and peptide exchange on MHC-I (Ilca et al., 2018a), we utilised these systems to specifically interrogate whether the intrinsic ability of MHC-I to interact with TAPBPR was significantly altered upon removal of the MHC-I N-linked glycan.

**Fig. 1:**
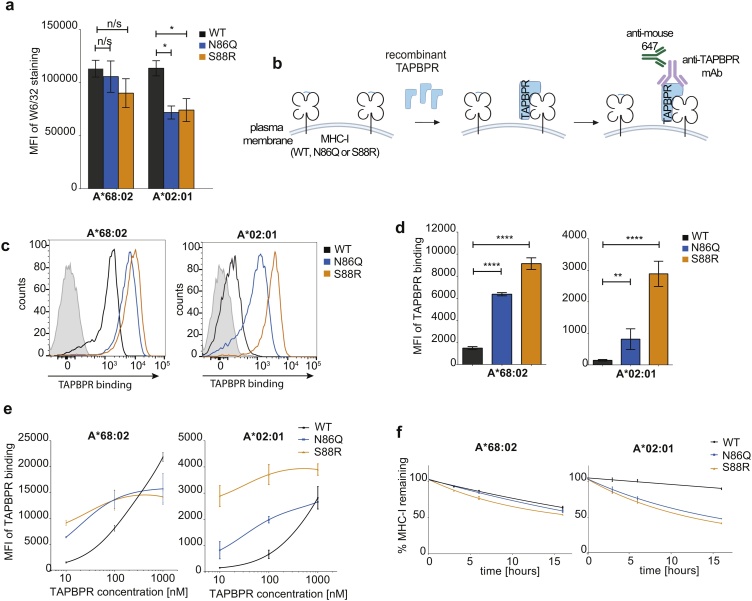
Non-glycosylated MHC-I display an enhanced ability to associate with TAPBPR. (**a**) Bar graph displaying MHC-I expressing (detected using W6/32) on IFN-γ stimulated HeLaM-HLA-ABC^KO^ cells reconstituted with HLA-A*68:02^WT^, HLA-A*02:01^WT^ or with their corresponding mutants, N86Q or S88R. (**b**) Schematic of the recombinant TAPBPR binding assay. (**c-e**) Binding of recombinant TAPBPR to HeLaM MHC-I cell panel stimulated with IFN-y. (**c**) Histograms and (**d**) bar graphs displaying the level of TAPBPR binding to the cells treated with 10 nM recombinant TAPBPR. In c, cells treated without TAPBPR are included as a negative control (solid grey lines). (**e**) Line graphs showing the binding of TAPBPR to the various MHC-I molecules following incubation with different concentrations of recombinant TAPBPR. (**f**) Line graphs showing the level of MHC-I remaining (detected with W6/32) on the cell line panel following treated with 10 μM BFA for the indicated time. In a, c, & d, error bars were generated based on SD from three independent experiments. In f, error bars are based on SD from triplicate samples within one experiment and the data is representative of two independent experiments. n/s = not significant, * = p < 0.05, ** = p < 0.01. **** = p < 0.0001.

To this end, HeLaM cells deficient in endogenous HLA-A, -B and -C expression were transduced with either wild-type MHC-I or MHC-I in which with NxS motif found at amino acid positions 86–88 was mutated either to QxS (N86Q) or to NxR (S88R), as previously described (Neerincx and Boyle, 2019a). We initially tested two MHC-I allotypes with different intrinsic abilities to associate with TAPBPR; HLA-A*68:02, the strongest TAPBPR binder across a wide panel of HLA, and HLA-A*02:01, a common HLA molecule with a decent level of binding to TAPBPR (Ilca et al., 2018a; Ilca et al., 2018b; Ilca et al., 2019; Neerincx and Boyle, 2019a). As found previously, removal of the glycan from HLA-A*02:01 reduced its surface expression to approximately 50 % of HLA-A*02:01^WT^, whereas for HLA-A*68:02, both mutants exhibited similar surface expression as the WT molecule (Fig. 1a) (Neerincx and Boyle, 2019a).

To test whether removal of the N-linked glycan from MHC-I impacted its interaction with TAPBPR, we incubated the cell line panel with recombinant human TAPBPR and quantified TAPBPR binding using flow cytometry (Fig. 1b). Our previous studies have demonstrated recombinant TAPBPR does not bind to IFN-γ treated HeLaM cells lacking HLA-A, -B and -C expression (Ilca et al., 2018a; Ilca et al., 2019). For both HLA-A*68:02 and HLA-A*02:01 expressing HeLaM cells, TAPBPR exhibited a significantly enhanced binding to both N86Q and S88R variants compared to the corresponding WT counterpart (Fig. 1c-e). For example, at 10 nM TAPBPR, the association of TAPBPR with HLA-A*02:01^S88R^ was >10-fold higher than with HLA-A*02:01^WT^, while a ∼5-fold increase in TAPBPR association with HLA-A*68:02^S88R^ was observed compared to HLA-A*68:02^WT^ (Fig. 1c-e). The increased binding of TAPBPR to non-glycosylated MHC-I could not be explained by changes in MHC-I expression, as the plasma membrane expression of the glycan deficient variants was either similar to WT molecules, in the case of HLA-A*68:02, or 50 % lower, in the case of HLA-A*02:01 (Fig. 1a). As an additional control, we tested the ability of the non-glycosylated MHC-I to bind to TAPBPR^TN5^, a TAPBPR mutant in which an isoleucine residue at position 261 was changed to a lysine resulting in a mutant unable to bind to MHC-I (Ilca et al., 2019; Hermann et al., 2013). Neither of the non-glycosylated forms for HLA-A*02:01 or HLA-A*68:02 showed any stable association with TAPBPR^TN5^, as similarly observed for the WT HLA counterparts (Figure S1a). This suggests that lack of the N-linked glycan does not result in additional stable interactions that do not typically occur between MHC-I and TAPBPR. While the binding of TAPBPR to the non-glycosylated MHC-I variants N86Q and S88R appeared to begin to saturate at 1 μM, this was not observed for WT glycosylated MHC-I (Fig. 1e).

### 3.2 Increased binding of TAPBPR to non-glycosylated MHC-I does not correlate with changes in MHC-I stability

As our plasma membrane assay utilising recombinant TAPBPR allows us to measure TAPBPR binding to modified MHC-I directly (Fig. 1b), it overcomes the direct impact that altering the glycan has on changing competing interactions for MHC-I otherwise observed in the complex intracellular environment; for example, a decrease in tapasin binding to MHC-I upon glycan removal which will subsequently increase TAPBPR binding (Neerincx and Boyle, 2019a; Hermann et al., 2013). However, although the results in Fig. 1 suggest that the affinity of the TAPBPR:MHC-I complex is significantly increased upon removal of the MHC-I glycan, an alternative explanation for this finding could be the presentation of lower affinity peptides on glycan-deficient MHC-I. This could result in an increased ability of recombinant TAPBPR to outcompete these lower-affinity peptides and subsequently form stable interactions with non-glycosylated MHC-I on the plasma membrane. However, neither WT nor non-glycosylated HLA-A*68:02 or -A*02:01 appeared peptide receptive when incubated with a low concentration of high affinity peptide (Figure S1b). We also tested the stability of peptide-MHC class I (pMHC-I) complexes present on the plasma membrane by performing brefeldin A (BFA) decay experiments on the panel of cell lines (Fig. 1f). The decay pattern of the MHC-I variants correlated with the relative differences in their expression levels at steady state (Fig. 1a). Namely, for HLA-A*68:02, no significant differences were observed in the decay rates between the WT molecule and the non-glycosylated variants (Fig. 1f). In contrast, the non-glycosylated HLA-A*02:01 showed a considerably higher rate of decay compared to the WT molecule (Fig. 1f). Thus, while the absence of the N-linked glycan resulted in a reduction in the molecular stability of HLA-A*02:01, it did not significantly affect HLA-A*68:02. Since both non-glycosylated HLA-A*68:02 variants displayed an enhanced ability to associate with TAPBPR (Fig. 1c-e), despite their highly similar molecular stability at the cell surface (Fig. 1f), our results suggest the MHC-I glycan directly influences the intrinsic interaction between TAPBPR and MHC-I.

### 3.3 MHC-I lacking a glycan exhibit an increased propensity to undergo peptide exchange at low concentrations of TAPBPR

**Fig. 2:**
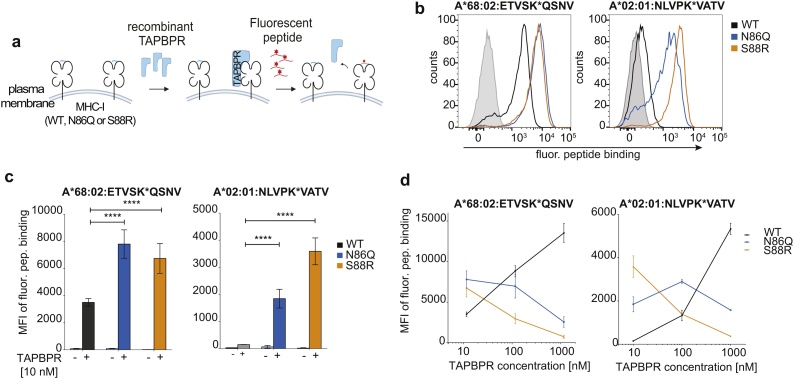
Non-glycosylated MHC-I display an enhanced susceptibility to TAPBPR-mediated peptide editing. (**a**) Schematic of the TAPBPR-mediated peptide binding assay. (**b-d**) Fluorescent peptide binding to the recombinant TAPBPR treated, IFN-γ stimulated HeLaM-HLA-ABC^KO^ cells reconstituted with either WT or non-glycosylated (N86Q and S88R) HLA-A*68:02 or HLA-A*02:01. (**b**) Histograms and (**c**) bar graphs displaying the level of ETVSK*QSNV and NLVPK*VATV binding to HLA-A*68:02 and HLA-A*02:01 variants, respectively, in the presence and absence of 10 nM recombinant TAPBPR, as indicated when cells were incubated with 10 nM fluorescent peptide (for 15 min to HLA-A*68:02 expressing cells and 60 min for HLA-A*02:01 expressing cells). In b, cells treated without peptide are included as negative control (solid grey lines). (**d**) Line graphs showing the binding of fluorescent peptide to the various MHC-I molecules following incubation with different concentrations of recombinant TAPBPR. Error bars were generated based on SD from three independent experiments. **** = p < 0.0001.

As the ability of MHC-I molecules to bind TAPBPR has been shown to correlate with their susceptibility to undergo peptide editing by TAPBPR (Ilca et al., 2018a; Ilca et al., 2019), we next tested whether the increased association between TAPBPR and non-glycosylated MHC-I translated into an increased ability to undergo TAPBPR-mediated peptide exchange. To this end, we incubated our cell line panel with MHC-I allotype-specific fluorescently-labelled peptides either in the presence or absence of recombinant TAPBPR then measured the level of bound peptide by flow cytometry (Fig. 2a) (Ilca et al., 2018a). The high-affinity fluorescent peptides used were NLVPK*VATV (specific for HLA-A*02:01) and ETVSK*QSNV (specific for HLA-A*68:02).

In the presence of 10 nM TAPBPR, the N86Q and S88R variants of both HLA-A*02:01 and HLA-A*68:02 exhibited a significant increase in TAPBPR-mediate peptide exchange resulting in high levels of exogenous peptide binding compared to their corresponding WT MHC-I counterpart (Fig. 2b-d). In contrast, in the absence of recombinant TAPBPR, exogenous fluorescent peptide binding to all cell lines was very low (Fig. 2c), confirming a lack of extensive passive exchange of endogenous peptides for exogenously added peptide in the absence of a catalyst in the time frame tested. Interestingly however, upon increasing concentrations of recombinant TAPBPR, while HLA-A*02:01^WT^ and HLA-A*68:02^WT^ exhibited a progressive increase in peptide exchange, as previously observed (Ilca et al., 2018a; Ilca et al., 2019), their respective non-glycosylated forms displayed an opposing effect (Fig. 2d). Namely, for HLA-A*02:01, at 10 nM TAPBPR concentration, the loading of NLVPK*VATV on HLA-A*02:01^WT^ was ∼10-fold and 20-fold lower than on HLA-A*02:01^N86Q^ and HLA-A*02:01^S88R^, respectively (Fig. 2c & d). However, at 1 μM TAPBPR, the level of peptide exchange on HLA-A*02:01^WT^ was >3-fold and >10-fold higher than on HLA-A*02:01^N86Q^ and HLA-A*02:01^S88R^, respectively (Fig. 2d). HLA-A*68:02 molecules showed a highly similar behaviour; at 10 nM TAPBPR, level of ETVSK*QSNV loaded onto HLA-A*68:02^WT^ was roughly half of the level observed on both HLA-A*68:02^N86Q^ and HLA-A*68:02^S88R^, while at 1 μM TAPBPR, the level of peptide exchange on HLA-A*68:02^WT^ was at least 5-fold higher than on both HLA-A*68:02^N86Q^ and HLA-A*68:02^S88R^ (Fig. 2c & d). Furthermore, a similar effect of TAPBPR was observed on the loading of a different HLA-A*02:01-specific peptide, YLLEK*LWRL, albeit with lower fluctuations in its binding across the different HLA-A*02:01 variants (Figure S2), potentially due to the higher affinity of this peptide compared to NLVPK*VATV (Ilca et al., 2018a).

The decline observed in peptide exchange on HLA-A*68:02 and -A*02:01 with increasing concentration of TAPBPR is suggestive of an enhancement in the competitive binding between TAPBPR and peptide to non-glycosylated MHC-I compared to their glycosylated forms. Thus, while the relatively moderate affinity of TAPBPR for naturally glycosylated MHC-I appears to result in a higher number of MHC-I undergoing peptide exchange with higher TAPBPR (catalyst) concentrations, we hypothesise that an increased affinity of TAPBPR for these non-glycosylated MHC-I enables TAPBPR to outcompete even high-affinity incoming peptide which would typically displace TAPBPR from glycosylated MHC-I.

### 3.4 TAPBPR exhibits an increased ability to dissociate high-affinity peptides from MHC-I molecules lacking a glycan

**Fig. 3:**
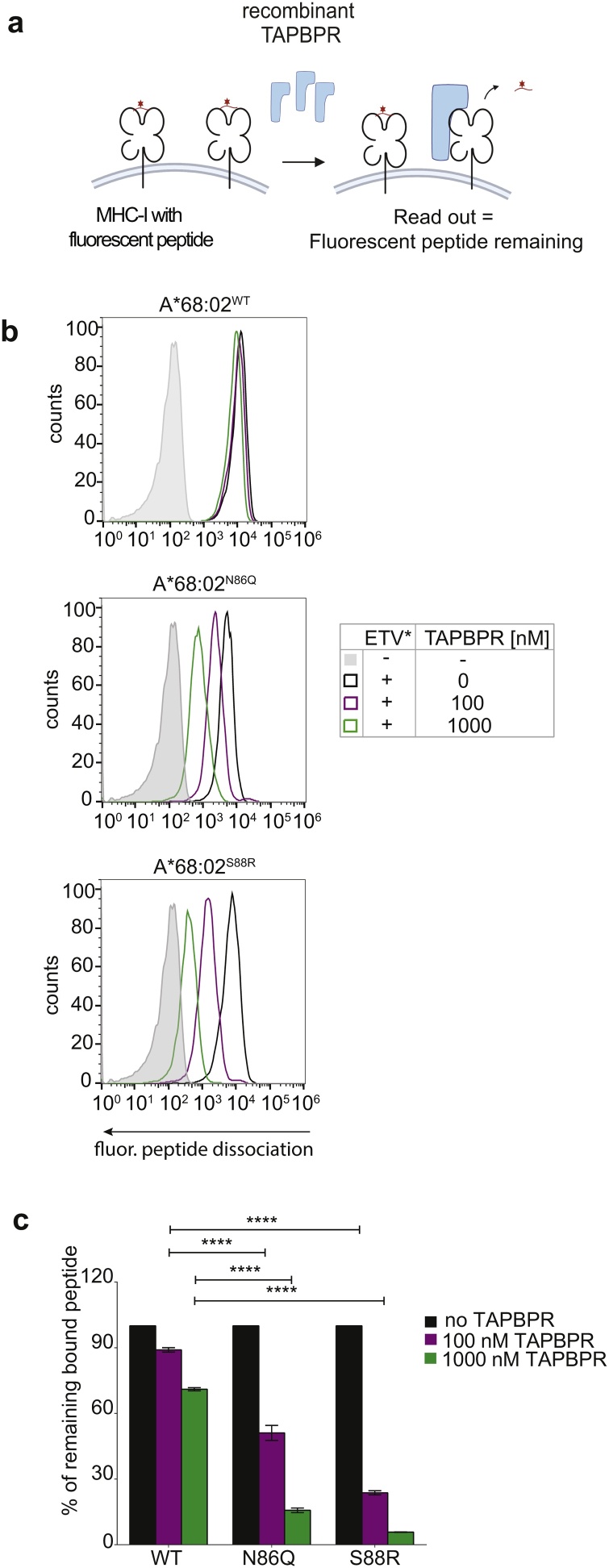
High affinity peptides are more easily dissociated from non-glycosylated MHC-I by TAPBPR. **(a)** Schematic of the TAPBPR-mediated MHC-I peptide dissociation assay. (**b)** Histograms and **(c**) bar chart showing the level of high affinity fluorescent peptide dissociation from HLA-A68:02 molecules by TAPBPR. To obtain similar levels of initial fluorescent peptide loading onto MHC-I, cells were treated with 10 nM ETVSK*QSNV for 15 min at 37 °C in the presence of either 100 nM TAPBPR (for HLA-A*68:02^WT^) or 10 nM TAPBPR (for HLA-A*68:02^N86Q^ and -A*68:02^S88R^). Following washing to remove unbound peptide and TAPBPR (see Figure S3c for remaining TAPBPR bound to cells following peptide loading), cells were incubated with 0, 100 nM or 1000 nM recombinant TAPBPR for 30 min and the dissociation ETVSK*QSNV was quantified by flow cytometry. In c, ETVSK*QSNV dissociation is depicted as % bound peptide upon addition of either 100 nM o 1000 nM TAPBPR compared to when no TAPBPR was added. Error bars represent SD based on three independent experiments. **** = p < 0.0001.

To test this hypothesis, we next measured whether TAPBPR could out-compete and dissociate high-affinity peptides from non-glycosylated MHC-I molecules present on the plasma membrane. TAPBPR-mediated peptide dissociation was assessed by loading the MHC-I molecules with a high-affinity fluorescent peptide in the presence of TAPBPR, washing the cells to remove any excess of unbound peptide and quantifying the decrease in bound fluorescent peptide upon addition of an excess of soluble TAPBPR (Fig. 3a).

Treatment of HLA-A*68:02^WT^ preloaded with ETVSK*QSNV with increasing concentrations of recombinant TAPBPR led to a low level of dissociation of the fluorescent peptide (Fig. 3b & c). In contrast, the treatment of HLA-A*68:02^N86Q^ or HLA-A*68:02^S88R^ preloaded with ETVSK*QSNV resulted in considerable dissociation of the fluorescent peptide in a dose-dependent manner (Fig. 3b & c). More specifically, at 100 nM, TAPBPR triggered the dissociation of ∼50 % of the pre-peptide loaded peptides from HLA-A*68:02^N86Q^ and ∼75 % of the peptides loaded onto HLA-A*68:02^S88R^, compared to just ∼10 % for HLA-A*68:02^WT^ (Fig. 3b & c). At 1 μM, TAPBPR dissociated ∼85 % and >90 % of the peptides loaded onto HLA-A*68:02^N86Q^ and HLA-A*68:02^S88R^, respectively, compared to ∼30 % of the peptide on HLA-A*68:02^WT^ (Fig. 3b & c).

To confirm that the increased ability of TAPBPR to dissociate peptides from non-glycosylated MHC-I molecules was not peptide specific, we measured the dissociation of YVVPFVAK*V (YVV*), a peptide with a higher relative affinity for HLA-A*68:02 than ETVSK*QSNV, in the presence of different TAPBPR concentrations (Figure S3a & S3b). While YVV* shows a much lower dissociation rate compared to ETV* in the presence of TAPBPR, the overall pattern of TAPBPR-mediated peptide dissociation was similar, with HLA-A*68:02^WT^ exhibiting <10 % peptide dissociation, even in the presence of 1 μM TAPBPR, while HLA-A*68:02^N86Q^ and HLA-A*68:02^S88R^ exhibited up to ∼30 % and ∼50 % loss of YVV*, respectively. Additionally, similar results were observed when measuring TAPBPR-mediated dissociation of NLVPK*VATV from HLA-A*02:01, with the caveat that the level of pre-loaded fluorescent peptide was lower for the S88R variant of HLA-A*02:01 than for either N86Q or WT variants (Figure S4a & S4b). Together, these data confirm the significantly enhanced peptide exchange ability of TAPBPR on non-glycosylated MHC-I molecules over naturally-occurring glycosylated ones.

### 3.5 TAPBPR is more resistant to peptide-mediated allosteric release from non-glycosylated MHC-I

**Fig. 4:**
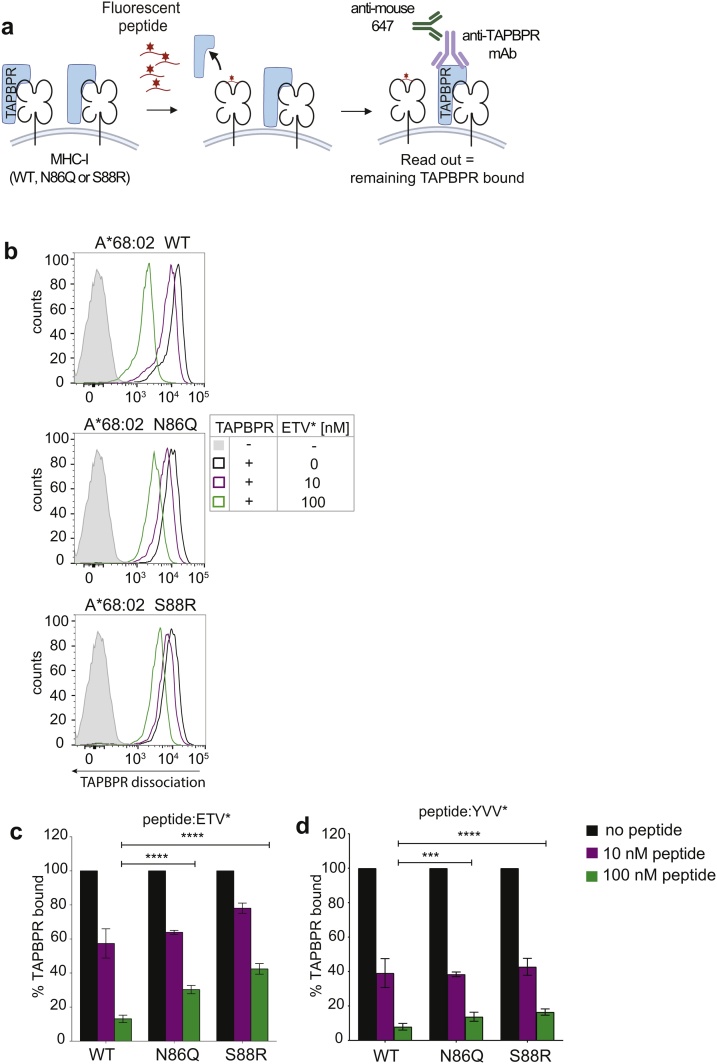
TAPBPR is more resistant to peptide-mediated allosteric release from non-glycosylated MHC-I. (**a**) Schematic of the TAPBPR dissociation assay. (**b**) Histograms and (**c & d**) bar graph depicting the level of TAPBPR dissociation from HLA-A*68:02 variants in the presence of increasing concentrations of peptide. IFN-γ stimulated HeLaM-HLA-ABC^KO^ cells reconstituted with the HLA-A*68:02 variants in were treated with 1 μM soluble TAPBPR for 30 min at 37 °C. Following washing to remove unbound TAPBPR, cells were incubated with either 0, 10 nM or 100 nM of (**b & c**) ETVSK*QSNV or (**d**) YVVPFVAK*V for 15 min. In c & d, TAPBPR dissociation is as depicted as % bound TAPBPR upon addition of either 10 nM or 100 nM peptide compared to when no peptide was added. Error bars represent SD based on three independent experiments. **** = p < 0.0001. *** = p 0.0001.

It has previously been shown that binding of high-affinity peptides to TAPBPR-bound peptide-receptive MHC-I triggers an allosteric release of TAPBPR from MHC-I (Morozov et al., 2016; Ilca et al., 2018a; McShan et al., 2018). Having confirmed the enhanced ability of TAPBPR to bind non-glycosylated MHC-I and to mediate peptide dissociation from these molecules, we next explored whether TAPBPR was more resistant to peptide-mediated allosteric release from MHC-I lacking an N-linked glycan. This would provide insight into whether the affinity of TAPBPR for MHC-I is dependent on the presence of the MHC-I glycan. To test the peptide-mediated displacement of TAPBPR from MHC-I, we treated the cell line panel with a high excess of recombinant TAPBPR, washed the cells to remove unbound TAPBPR and subsequently measured TAPBPR dissociation in the presence of different concentrations of high-affinity peptide (Fig. 4a). Consistent with data shown previously (Fig. 1e), similar levels of TAPBPR bound to the glycosylation variant of HLA-A*68:02 when 1 μM recombinant TAPBPR was added to cells (Fig. 4b – black line). Upon subsequent incubation with ETVSK*QSNV, a peptide with high affinity for HLA-A*68:02, we observed a considerably higher degree of TAPBPR dissociation from HLA-A*68:02^WT^ (∼85 %), than from both HLA-A*68:02^N86Q^ (∼70 %) and HLA-A*68:02^S88R^ (∼60 %) (Fig. 4b & c). Furthermore, our findings demonstrated that while the same hierarchy of TAPBPR displacement from HLA-A*68:02 molecules was observed using YVVPFVAK*V, this peptide which has higher affinity HLA-A*68:02 than ETVSK*QSNV, was able to dissociate more TAPBPR from the HLA-A*68:02 molecules than ETVSK*QSNV (∼92 % TAPBPR displacement from WT, ∼86 % from N86Q and ∼83 % from S88R) (Fig. 4d). Furthermore, a similar TAPBPR dissociation profile, albeit with difference in relative amounts, was also observed using the HLA-A*02:01 variants with the HLA-A2 binding peptide NLV* (Figure S5). Together, these findings show that the same amount of high-affinity peptide manages to out-compete less TAPBPR from non-glycosylated MHC-I compared to WT MHC-I carrying a mature glycan. Given that TAPBPR seems more resistant to peptide-mediated allosteric release from MHC-I in the absence of an N-linked glycan, TAPBPR appears to have a higher affinity for non-glycosylated MHC-I than molecules carrying a mature glycan.

### 3.6 Even HLA-B*27:05, a poor TAPBPR binder, displays enhanced TAPBPR-mediated peptide editing in the absence of an N-linked glycan

**Fig. 5:**
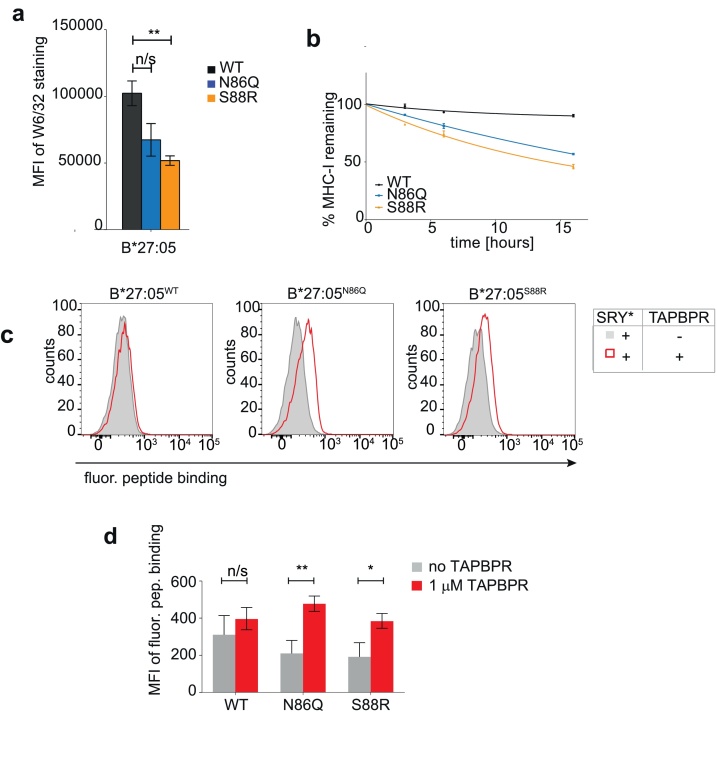
Non-glycosylated HLA-B*27:05 also displays an enhanced susceptibility to TAPBPR-mediated peptide editing. (**a**) Bar graph displaying MHC-I expression (detected using W6/32) on IFN-γ stimulated HeLaM-HLA-ABC^KO^ cells reconstituted with HLA-B*27:05^WT^, -B*27:05^N86Q^ or –B*27:05^S88R^. (**b**) Line graph showing the level of MHC-I remaining (detected with W6/32) on the HLA-B*27:05 cell line panel following treated with 10 μM BFA for the indicated time. (**c**) Histograms and (**d**) bar graph displaying the level of SRYWK*IRTR (SRY*) binding to the HLA-B*27:05 variants in the presence and absence of 1 μM recombinant TAPBPR. In a and d, error bars were generated based on SD from three independent experiments. In b, error bars are based on SD from triplicate samples within one experiment and the data is representative of two independent experiments. n/s = not significant, * = p < 0.05, ** = p < 0.01.

Finally, we tested whether removing the glycan from HLA-B*27:05, an MHC-I molecule considered a poor TAPBPR binder (Ilca et al., 2018b; Ilca et al., 2019; Neerincx and Boyle, 2019a), would also increase its susceptibility to TAPBPR-mediated peptide exchange. To this end, HeLaM cells deficient in endogenous HLA-A, -B and -C expression were transduced with either HLA-B*27:05^WT^, -B*27:05^N86Q^ or –B*27:05^S88R^ (Neerincx and Boyle, 2019a). As found previously (Neerincx and Boyle, 2019a), removal of the glycan from HLA-B*27:05 reduced its surface expression to approximately 50 % compared to HLA-B*27:05^WT^ (Fig. 5a). Furthermore, BFA decay assays revealed glycosylation mutants of HLA-B*27:05 showed a considerably higher decay rates compared to HLA-B*27:05^WT^ (Fig. 5b). We were unable to detect any obvious binding of recombinant TAPBPR to any of the plasma membrane expressed HLA-B*27:05 variants (Figure S6). However, our previous findings showed that TAPBPR does not necessarily require stable association to MHC-I in order to mediate peptide exchange (Ilca et al., 2018b; Ilca et al., 2019). When we subsequently tested the ability of TAPBPR to mediate peptide exchange on the HLA-B*27:05 variants, the glycosylation mutants of HLA-B*27:05 exhibited an increased susceptibility to TAPBPR-mediated peptide exchange (Fig. 5c & d). Specifically, recombinant TAPBPR was unable to load any significant amounts the HLA-B*27:05-specific fluorescently-labelled peptide SRYWK*IRTR onto HLA-B*27:05^WT^ (Fig. 5c & d). In contrast, in the presence of recombinant TAPBPR, a low but significant enhancement in the level of exogenous peptide loading was observed on both HLA-B*27:05^N86Q^ and HLA-B*27:05^S88R^, compared to when cells were treated with peptide alone (Fig. 5c & d). These results confirm that N-linked glycosylation on MHC-I plays an important role in modulating its interaction with TAPBPR and consequently their susceptibility to peptide editing.

## 4 Discussion

Here, we reveal the interaction between TAPBPR and MHC-I is stronger when MHC-I lacks its naturally-present N-linked glycan. We found that the glycosylation status of MHC-I molecules subsequently influences their ability to undergo TAPBPR-mediated peptide exchange. For HLA-B*27:05, an MHC-I molecule which binds relatively weakly to TAPBPR (Ilca et al., 2019), we observed an increased propensity of the non-glycosylated variant to undergo peptide exchange in the presence of high TAPBPR concentrations compared to glycosylated HLA-B2*27:05. However, for MHC-I allotypes which bind strongly to TAPBPR, *i.e.* those belonging to the HLA-A2 and –A24 superfamily (Ilca et al., 2019), the picture is more complex. In the presence of low concentrations of TAPBPR, non-glycosylated variants of both HLA-A*68:02 and HLA-A*02:01 exhibit an increased propensity to undergo TAPBPR-mediated peptide exchange compared to their glycosylated counterparts. This is likely reflective of the increased affinity of TAPBPR for those non-glycosylated MHC-I variants, enabling it to more effectively outcompete the endogenous peptides presented on those molecules at the cell surface compared to their wild-type counterparts. Consistent with this idea, with increasing TAPBPR concentrations, we observed a progressive reduction in the level of incoming high-affinity peptides that remained bound to the non-glycosylated molecules at equilibrium. This suggested that due to its increased affinity for non-glycosylated over glycosylated MHC-I molecules, TAPBPR can even compete with high-affinity peptides for the binding to the non-glycosylated variants; consequently, titrating TAPBPR in the peptide-exchange reaction shifts the equilibrium from the fluorescent peptide-bound state of the non-glycosylated MHC-I towards their TAPBPR-bound state. In contrast, due to the relatively lower affinity of TAPBPR for the glycosylated forms of these MHC-I allotypes, increasing concentrations of TAPBPR could only enhance the peptide exchange rate, as the chaperone can only dissociate endogenous peptides from those MHC-I molecules and would in turn be strongly and irreversibly outcompeted by the incoming high-affinity peptides. Confirming this theory, we subsequently found that a high excess of TAPBPR dissociated peptides of high affinity more easily from the non-glycosylated variants of HLA-A*68:02 and HLA-A*02:01 than from their corresponding glycosylated counterparts. Furthermore, TAPBPR was more resistant to peptide-mediated allosteric release from non-glycosylated HLA-A*68:02 and -A*02:01 compared to species with a glycan attached.

On a molecular level, how might removal of the N-linked glycan increase the affinity between TAPBPR:MHC-I? Given that the glycan attached to MHC-I is important for its association with calreticulin, and consequently with tapasin, one possibility could be non-glycosylated MHC-I containing lower affinity peptides. While our evidence suggests that non-glycosylated HLA-A*02:01 molecules had a faster BFA decay, therefore presumably contained lower affinity peptides, this was not the case for HLA-A*68:02 molecules (Fig. 1f). However, non-glycosylated variants of both these HLA-A molecules appeared to exhibit a higher affinity for TAPBPR compared to their glycosylated counterparts. Furthermore, if the increased binding observed was simply due to a change in the affinity of MHC-I cargo, this would not explain the findings we observed in Fig. 3, namely that TAPBPR is able outcompete the same high affinity fluorescent peptides from the non-glycosylated MHC-I but not from the glycosylated forms; nor would it explain the reduction in ability of high-affinity peptides to release TAPBPR from non-glycosylated MHC-I compared to their glycosylated counterparts as observed in Fig. 4.

Another potential explanation is that a bulky glycan on MHC-I poses some degree of steric hindrance on the TAPBPR:MHC-I interaction. Indeed, crystal structures of the TAPBPR with non-glycosylated MHC-I complex have revealed residue Y84 on MHC-I, near to N86, to contribute to the interaction face (Thomas and Tampe, 2017; Jiang et al., 2017). Interestingly, it has recently been suggested that there is a direct interaction between tapasin and MHC-I involving the N86-linked MHC-I glycan and residues in the vicinity of the tapasin 11–20 loop (Fisette et al., 2020). Like the homologous K22-D35 loop of TAPBPR, the tapasin 11–20 loop has been proposed to play a role in peptide selection (Ilca et al., 2018b; Sagert et al., 2020; Hafstrand et al., 2019). In the case of tapasin, a study based on molecular dynamics simulations proposed that the presence of the MHC-I glycan causes steric hindrance on the interaction with tapasin, consequently pushing the tapasin loop region toward the MHC-I F pocket (Fisette et al., 2020). Whether a similar mechanism occurs for TAPBPR remains to be determined.

The discovery that the glycan attached to MHC-I significantly influences the affinity of its interaction with TAPBPR has important implications, on both an experimental level and in a biological context. Experimentally, while the use of non-glycosylated MHC-I likely assisted with the crystallisation of the TAPBPR:MHC-I complex (Thomas and Tampe, 2017; Jiang et al., 2017), it is possible that naturally-glycosylated MHC-I may adopt slightly different interaction sites with TAPBPR or even alternative conformations when bound to TAPBPR. Thus, our understanding of the mechanisms of peptide editing by TAPBPR would be further elucidated if it was possible to capture TAPBPR with MHC-I in its natural glycosylated state. Furthermore, we must be mindful that both affinity of TAPBPR for MHC-I and its efficiency to perform peptide exchange is altered in the presence and absence of the glycan attachment on MHC-I when both designing assays and comparing the results of different experimental systems. While the current assays used to explore TAPBPR-mediated peptide editing have used either bacterially expressed MHC-I which lack glycan attachment (Hermann et al., 2015; Morozov et al., 2016; Sagert et al., 2020; Neerincx et al., 2017) or plasma membrane expressed MHC-I which has a complex, mature oligosaccharide attached (Ilca et al., 2018a; Ilca et al., 2018b; Ilca et al., 2019), to date, the assays currently used to probe TAPBPR-mediated peptide exchange are at either extremes in terms of MHC-I oligosaccharide attachment and likely do not fully represent the forms of MHC-I for which TAPBPR naturally functions on.

Our findings also have a number of biological implications. Oligosaccharide attachment to glycoproteins undergoes significant changes as the molecules traffic through the secretory pathway. Our findings here suggest that the size and composition of the glycan attached to MHC-I could significantly impact the ability of TAPBPR to bind and consequently function on MHC-I *in vivo*. While it is presumably unlikely that TAPBPR functions on MHC-I totally devoid of a glycan attachment *in vivo*, there may well be a “sweet spot” in the secretory pathway, based on the glycosylation status of MHC-I, that maximises the ability of TAPBPR to function as a peptide editor. Our data also suggests that the glycosylation status of MHC-I has the potential to influence the function of TAPBPR. For example, TAPBPR may perform a chaperoning role on some MHC-I species to which is exhibits a higher affinity but then flip to an efficient peptide editor as the MHC-I glycan undergoes processing, such as in the ERGIC or Golgi apparatus. This may also be applicable to alternative ligands for TAPBPR, such as MR1 (McWilliam et al., 2020). Furthermore, the glycosylation status of MHC-I could assist in the release of TAPBPR, perhaps with larger or more complex sugar attachments reducing its affinity for TAPBPR as the MHC-I molecule traffics towards the plasma membrane. Our work also suggests that the local concentration of TAPBPR, in combination with variation in the glycan attached to MHC-I, may influence the functional outcome of the interaction between these two molecules. Undoubtedly, further research is needed to gain full insight regarding the impact that the MHC-I glycosylation status has in shaping the repertoire of peptides presented.

## Funding source

This work was supported by 10.13039/100010269Wellcome [grant numbers 109076/Z/15/A and 219479/Z/19/Z].

## CRediT authorship contribution statement

**F. Tudor Ilca:** Conceptualization, Methodology, Formal analysis, Investigation, Writing - original draft, Writing - review & editing. **Louise H. Boyle:** Conceptualization, Formal analysis, Writing - original draft, Writing - review & editing, Supervision, Funding acquisition.
