# Supplementary material for: The glycosylation status of MHC class I molecules impacts their interactions with TAPBPR
Source: Mol Immunol. 2021 Nov;139:168–76. doi: 10.1016/j.molimm.2021.09.007 (PMC8524320; doi:10.1016/j.molimm.2021.09.007)
Supplement: Supplementary file 1 [file mmc1.docx]

**Supplementary figures:**

**Figure S1: Non-glycosylated MHC-I do not bind to TAPBPR^TN5^ and do not appear to be peptide receptive in the presence of low concentrations of high affinity peptide.** IFN-γ stimulated HeLaM-HLA-ABC^KO^ cells reconstituted with the WT, N86Q or S88R variants of either HLA-A*68:02 or HLA-A*02:01 were incubated with (**a**) 1 μM soluble TAPBPR^TN5^ for 30 min at 37°C or (**b**) 10 nM ETVSK*QSNV for 15 min at 37^o^C for HLA-A*68:02 expressing cells and 10 nM NLVPK*VATV for 60 min at 37^o^C for HLA-A*02:01 variants. Binding of TAPBPR or peptide to the surface of cells was quantified by flow cytometry. Histograms are representative of three independent experiments.

**Figure S2: The enhanced susceptibility of non-glycosylated HLA-A*02:01 to TAPBPR-mediated peptide editing is also observed with a second HLA-A*02:01 binding peptide**

(**a**) Histograms displaying the level of YLLEK*LWRL binding to the HLA-A*02:01 variants in the presence of 10 nM recombinant TAPBPR. Cells treated without peptide are included as negative control (solid grey lines). (**b**) Bar graph summarising the binding of YLLEK*LWRL binding to the HLA-A*02:01 variants in the absence and presence of 10 nM recombinant TAPBPR. (**c**) Line graphs showing the binding of YLLEK*LWRL to the HLA-A*02:01 variants following incubation with different concentrations of recombinant TAPBPR. Error bars were generated based on SD from three independent experiments. ****= p<0.0001.

**Figure S3: Enhanced dissociation of a second high affinity peptide from non-glycosylated HLA-A*68:02 by TAPBPR**

(**a)** Histograms and **(b**) bar chart showing the level of YVVPFVAK*V dissociation from HLA-A*68:02 molecules by TAPBPR. To obtain similar levels of initial fluorescent peptide loading onto MHC-I, cells were treated with 10 nM YVVPFVAK*V for 15 min at 37°C in the presence of either 100 nM TAPBPR (for HLA-A*68:02^WT^) or 10 nM TAPBPR (for HLA-A*68:02^N86Q^ and -A*68:02^S88R^). (**c**) Histograms show the remaining TAPBPR bound to HLA-A*68:02 expressing HeLaM cells treated with either YVVPFVAK*V or ETVSK*QSNV following washing to remove unbound peptide and TAPBPR. **(a & b)** Cells were subsequently incubated with 0, 100 nM or 1000 nM recombinant TAPBPR for 30 min and the dissociation YVVPFVAK*V was quantified by flow cytometry. In b, YVVPFVAK*V dissociation is depicted as % bound peptide upon addition of either 100 nM o 1000 nM TAPBPR compared to when no TAPBPR was added. Error bars represent SD based on three independent experiments. ***=P<0.001, ****= p<0.0001

**Figure S4: Enhanced dissociation of a high affinity peptide from non-glycosylated HLA-A*02:01 by TAPBPR**

(**a)** Histograms and **(b**) bar chart showing the level of NLVPK*VATV dissociation from HLA-A*02:01 molecules by TAPBPR. To obtain similar levels of initial fluorescent peptide loading onto MHC-I, cells were treated with 10 nM NLVPK*VATV for 15 min at 37°C in the presence of either 100 nM TAPBPR (for HLA-A*02:01^WT^) or 10 nM TAPBPR (for HLA-A*02:01^N86Q^ and -A*02:01^S88R^). (**c**) Histograms show the remaining TAPBPR bound to HLA-A*02:01 expressing HeLaM cells following washing to remove unbound peptide and TAPBPR. **(a & b)** Cells were subsequently incubated with 0, 100 nM or 1000 nM recombinant TAPBPR for 30 min and the dissociation NLVPK*VATV was quantified by flow cytometry. In b, NLVPK*VATV dissociation is depicted as % bound peptide upon addition of either 100 nM o 1000 nM TAPBPR compared to when no TAPBPR was added. Error bars represent SD based on three independent experiments. n/s = not significant, ***=P<0.001, ****= p<0.0001.

**Figure S5- Dissociation of TAPBPR from HLA-A*02:01.**

(**a**) Histograms and (**b**) bar graph depicting the level of TAPBPR dissociation from HLA-A*02:01 variants in the presence of increasing concentrations of peptide. IFN-γ stimulated HeLaM-HLA-ABC^KO^ cells reconstituted with the HLA-A*02:01 variants were treated with 1 μM soluble TAPBPR for 30 min at 37°C. Following washing to remove unbound TAPBPR, cells were incubated with either 0, 10 nM or 100 nM NLVPK*VATV for 15 min. In b, TAPBPR dissociation is as depicted as % bound TAPBPR upon addition of either 10 nM o 100 nM ETVSK*QSNV compared to when no peptide was added. Error bars represent SD based on three independent experiments. n/s = not significant, ****= p<0.0001.

**Figure S6 - Mutations of the N-glycosylation motif in HLA-B*27:05 do not obviously enhance TAPBPR binding.** IFN-γ stimulated HeLaM-HLA-ABC^KO^ cells reconstituted with the WT, N86Q or S88R variant of HLA-B*27:05 were incubated with 1 μM soluble TAPBPR for 30 min at 37°C. Binding of TAPBPR to the surface of cells was quantified by flow cytometry. Histograms are representative of three independent experiments.
